# Direct quantification of rare earth doped titania nanoparticles in individual human cells

**DOI:** 10.1088/0957-4484/27/28/285103

**Published:** 2016-06-03

**Authors:** J C G Jeynes, C Jeynes, V Palitsin, H E Townley

**Affiliations:** 1 Wellcome Trust Biomedical Modelling and Analysis Centre, University Of Exeter, UK; 2 Surrey Ion Beam Centre, University of Surrey, UK; 3 Nuffield Department of Obstetrics & Gynaecology, Women’s Centre, John Radcliffe Hospital, Oxford, OX3 9DU, University of Oxford, UK; 4 Department of Engineering Sciences, Parks Road, Oxford, OX1 3PJ, UK; c.jeynes@exeter.ac.uk

**Keywords:** ion beam analysis, proton induced x-ray emission, lanthanides, nanoparticles, human cells

## Abstract

There are many possible biomedical applications for titania nanoparticles (NPs) doped with rare earth elements (REEs), from dose enhancement and diagnostic imaging in radiotherapy, to biosensing. However, there are concerns that the NPs could disintegrate in the body thus releasing toxic REE ions to undesired locations. As a first step, we investigate how accurately the Ti/REE ratio from the NPs can be measured inside human cells. A quantitative analysis of whole, unsectioned, individual human cells was performed using proton microprobe elemental microscopy. This method is unique in being able to quantitatively analyse all the elements in an unsectioned individual cell with micron resolution, while also scanning large fields of view. We compared the Ti/REE signal inside cells to NPs that were outside the cells, non-specifically absorbed onto the polypropylene substrate. We show that the REE signal in individual cells co-localises with the titanium signal, indicating that the NPs have remained intact. Within the uncertainty of the measurement, there is no difference between the Ti/REE ratio inside and outside the cells. Interestingly, we also show that there is considerable variation in the uptake of the NPs from cell-to-cell, by a factor of more than 10. We conclude that the NPs enter the cells and remain intact. The large heterogeneity in NP concentrations from cell-to-cell should be considered if they are to be used therapeutically.

## Introduction

Nanoparticles (NPs) offer unique capabilities and applications to healthcare, ranging from therapeutics to diagnostics. Doping NPs with rare earth elements (REEs) has previously been used to enhance the effect of x-rays on cancer cells [1, 2], and has many other interesting biosensing applications. The doped titania NPs used in this work were designed for use as radiosensitizers, to improve the efficacy of radiotherapy. Titania NPs can be excited by UV light and are used for photodynamic therapy for surface cancer treatments, but due to the penetration depth of UV light, deep tissues or large tumours cannot be treated by this method. The addition of the REE dopants extend the range of excitation to x-ray energy [3].

Gadolinium is used to dope the titania because of its high photon interaction cross-section, and Europium and Erbium were combined to include smaller edge absorption features around the main absorption peak. Titanium oxide is an ideal host for high mass elements such as rare earths because x-rays and electrons when absorbed increase reactive oxygen species that occur at the titanium oxide NP surface. For the clinical use of this radiosensitisation method, it is important to investigate the integrity of the NPs in human cells and test if REEs are leaching from the titania matrix.

There have been some studies investigating the integrity of a variety of NPs under physiological conditions. Toxicity tests (e.g. cell death assays) showed that rare earth oxide NPs (including Gd, Eu, La, Ce) readily dissociated in the lysosomes of macrophages, releasing rare earth ions into the cytoplasm. This triggered a number of inflammatory responses, fibrogenesis and cell death [3]. Similarly, quantum dots made of CdSe have been shown to disintegrate in the acidic conditions of the lysosomes [4].

The REE-doped titania NPs used for our study have previously been tested using a variety of toxicity assays [1, 2]. The radiosensitizing NPs are known to enter human cells by endocytosis, are sequestered within lysosomes of cells and in the absence of irradiation are non-toxic to breast cancer cells, and show no off-target toxicity in mice xenograft models at the concentrations tested. However, it is still unknown whether any of the REEs leach out of the NP matrix. There are a number of studies discussing the site of REE dopants within the titania crystal, but it remains unproven whether they replace Ti in substitutional sites or are incorporated at interstitial sites [5].

There is precedent for the use of gadolinium in medical imaging where gadolinium chelates are commonly used to improve tissue contrast in MRI. However, gadolinium is extremely toxic in its unbound state, with considerable cardiovascular and neurological toxicity. At clinical levels, gadolinium-based contrast agents are safe in patients with normal kidney function, but administration to patients with renal dysfunction can result in nephrogenic systemic fibrosis leading to disabling contractures and even death [6]. It appears to be unlikely that the structural integrity of the NPs would be compromised within the body, however, to satisfy concerns with regards to possible contra-indications it is important for future work to determine the localisation of the REEs with regards the doped titania NPs.

There are a variety of analytical techniques used to investigate NP integrity, each having different limitations. Bulk elemental analysis techniques like inductively coupled mass spectrometry and atomic emission spectroscopy, average the content across thousands of cells and can mask the heterogeneity of individual cells. If the NPs are to be used therapeutically within a tumour, then cell-to-cell heterogeneity may be an important criterion to determine. Moreover, bulk analysis does not co-localise elements spatially, which is crucial for determining if an element has leached out of the matrix material of the NP. Electron microscopy [7], x-ray fluorescence [8, 9] and flow cytometry have all been used to study NPs in single cells (for a review see [10]) with each technique limited in some respect. For instance, transmission electron microscopy requires ultra-thin sections of the cells, giving limited field of view (in *X*, *Y* and *Z* planes) and generally poor counting statistics.

Here, we have used a proton microprobe with micron resolution to co-localise and map elements spatially. This technique has excellent sensitivity to both light and heavy elements at ppm concentrations, has a large field of view, and does not require cells to be sectioned. Nuclear microprobes have a long history of elemental mapping of biological tissues and cells on a micron scale with high sensitivity and accurate quantification available when x-ray and particle spectra are used self-consistently [11, 12]. Previously, we have used this technique to measure the absolute number of gold NPs in cancer cells [13] as well as the concentration of cis-platin within the nucleus of individual cells [14].

In the current work, we show that the concentration of dopant REEs in titania NPs is the same both inside and outside the cells, and are spatially distributed with the titania matrix. We discuss the limitations of measurement and conclude that the NPs remain intact within the cell. Moreover, we show that there is a large cell-to-cell variability in the NP concentration.

## Methods

### Cell culture

HeLa cells (a human cervical cancer cell line) were used for these experiments, as uptake of NPs is high with these cells. HeLa cells were grown in Eagle minimal essential media ((EMEM) Lonza, Wokingham, UK) with 10% foetal bovine serum, 2 mM glutamine and 100 IU ml^−1^ penicillin and 0.1 mg ml^−1^ streptomycin incubated at 37 °C with 5% CO_2_.

### NP synthesis and preparation

The NPs were prepared and analysed as described previously [2]. Briefly, the rare earth metals (Gd, Er, Eu) were suspended in titanium isopropoxide at 1 mol%. This solution was then added to a 50/50 water/isopropanol mix while stirring vigorously. The precipitate was washed, collected by filtration and autoclaved. The slurry was dried and ground to a fine powder before firing. The NPs were then coated with silica by adding 3-Mercaptopropyltrimethoxy-silane and sodium silicate followed by washing and finally re-suspension in deionised water. In solution the NPs are polycrystalline in nature (anatase) with a particle size of approximately 60 nm and with a primary crystallite of 3–5 nm.

### Sample preparation

Here, 1 × 10^5^ cells were seeded on custom made 35 mm diameter dishes lined with polypropylene film (4 *μ*m thick), fibronectin coated (1 mg ml^−1^ in distilled water) and left to attach overnight. The next day, the cells were incubated with titania NPs for 4 h in EMEM media, then washed in phosphate buffered saline (PBS), and finally fixed with 4% paraformaldehyde.

For microprobe analysis preparation, the cells were washed again with PBS, dehydrated in a series of ethanol dilutions and finally air dried in hexamethyldisilazane (HMDS).

### Proton microprobe setup

The samples are placed into the vacuum chamber of a 2 MV tandem accelerator [13, 15]. A 2.5 MeV proton beam was focused to a 2 *μ*m spot size of about 300 pA. At this energy, the protons can penetrate the entire thickness of human cells (about 6 *μ*m) with very little energy loss. The samples were scanned in 200 × 200 *μ*m regions for 5 h, with each region containing 10–30 cells. The energy spectrum of photons or scattered particles from each pixel is recorded (with OMDAQ) so that the data can be reconstructed and analysed offline using OMDAQ to manipulate the pixel data, and the DataFurnace code for quantification following the methods given in detail in [13].

### Ion beam analysis

The details of the analysis procedure have been described in detail previously for single cells [13]. Briefly, using the signals from the proton induced x-ray emission (PIXE) detector the cells are imaged (particularly using the phosphorus signals) (see figures 2 and 3), and then can be quantitatively measured using the combined PIXE and elastic backscattered (EBS) signal using DataFurnace [16]. For clarity, we use the term ‘elastic backscattering’, since the proton scattering cross-sections are mostly non-Rutherford (a clear description of the EBS-RBS difference as well as analysis details can found in the supporting information).

The PIXE and EBS spectrum from each cell, or any region of interest, can be isolated and analysed individually. As the irradiated area for each selected cell is precisely known, the areal density units are converted into units of mass (see table 1).

**Table 1.** Rare earth content of titania nanoparticles.

Sample	Region	Ti	Er	Gd	Eu	Rare earths (REE)		
						Sum	Ti/REs	Uncertainty
		TFU	TFU	TFU	TFU	TFU		
#77	On substrate	4.49	0.0071	0.0305	0.0158	0.0533	84.24	16%
#77	In cell	25.44	0.0849	0.0927	0.1259	0.3035	83.82	45%
#73	On substrate	3.16	0.0151	0.0185	0.0169	0.0505	62.57	12%
#73	In cell	40.62	0.1964	0.2329	0.1934	0.6227	65.23	3%

Note. TFU (‘thin film units’) means 10^15^ atom cm^−2^. The molar % (number of atoms) of rare earth relative to Ti in the nanoparticles is highlighted in bold in table 1, with data obtained from the fitted EBS spectra shown in figures 2 and 3. The uncertainty on the REE quantification is mostly from overlap uncertainties from trace levels of transition metal (e.g. Fe, Cu) and K lines interfering with the L lines of the REE. The comparison (ratio) of in-cell and on substrate values of the Ti/REE ratios for #77, #73 is, respectively, 1.00 and 1.05. That is, the NPs in the cells suffer no degradation.

PIXE data *alone* are used when (1) the counts are high (>1000 per element) and (2) the absolute values are not needed, and a ratio will suffice (see figure 4(d)). EBS/PIXE data are used together when (1) the counts are low (<200) and (2) absolute concentrations of elements are required (see table 1: it is worth noting the analysis is much more involved and time-consuming than PIXE analysis alone).

## Results

**Figure 1.**
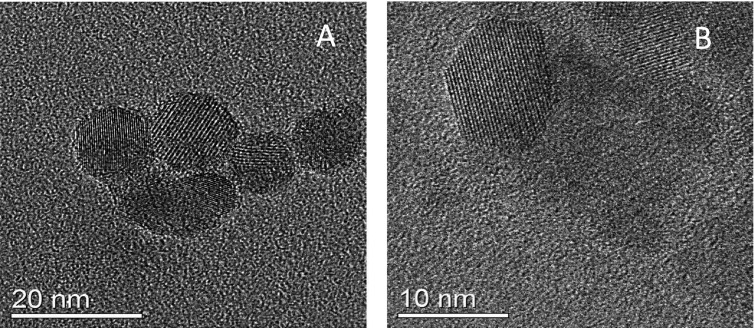
Transmission electron micrographs of primary crystallite titania NPs. (A) Titania NPs only (B) Titania NPs doped with 1% Gd. The titania lattice structure can be seen clearly in both samples. However, the Gd atoms could not be localised in the structure.

Figure 1 shows TEM images of primary crystallite titania NPs. We show the primary crystals because the crystal lattice can clearly be seen. The images show a truncated tetragonal bipyramid shape (anatase). Figure 1(A) shows an image of titania NPs which have not been REE-doped. Figure 1(B) shows titania NPs that have been doped with 1% Gd. A very similar titania lattice structure can be seen as in figure 1(A). However, it was impossible to detect any visual (or spectroscopic) signs of the Gd atoms. The apparent shape of the NP depends on how it is lying on the grid and so can appear round or hexagonal with orientation.

**Figure 2.**
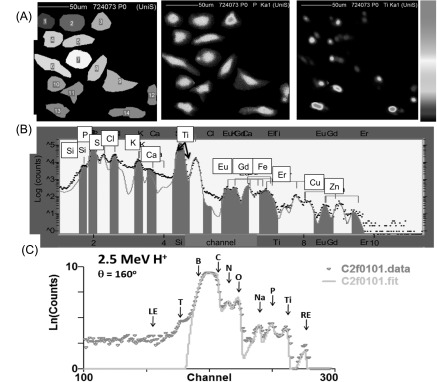
The figure shows an example of a region of HeLa cells containing titania nanoparticles (doped with 1 mol% each of Gd, Eu and Er), scanned with a proton microprobe. (A) (*left image*) A mask over each individual cell visualised using the phosphorus signal (*middle image*) and the titanium signal (*right image*). The scale bar is linear and shows counts per pixel with a maximum of about 10^4^ counts. (B) The proton induced x-ray emission (PIXE) signal of all the detectable elements from the combined signal from all cells (C). Elastic backscattered spectrum (EBS) of C, N, O, Na, P, Ti and the rare earth group (‘RE’: Er, Gd, Eu). The carbon signal comes from both the cells and the 4 *μ*m polypropylene foil, and the back edge signal of the foil (‘B’) is shown. ‘T’ is an unfitted signal to do with the thickness of the cells, and ‘LE’ is another unfitted signal coming from stray beam scattered in transmission.

Figure 2 shows images and spectrum from a 200 *μ*m scanned region of cells containing REE-doped NPs. Here, we have created masks over the cells so that the PIXE and EBS spectrum shown comes from the combined signal only of those cells, excluding signals that come from in-between the cells. The PIXE spectrum shows peaks from all major and trace elements of the cells (e.g. phosphorus, sulphur, potassium, copper, zinc) and the NPs inside the cells (Ti, Gd, Er, Eu). The peaks are fitted using GUPIX software (see Methods and supporting information) that gives concentrations of the elements in ng cm^−2^, but the accuracy can be compromised by sample thickness uncertainties, important for x-ray absorption profiles within the sample. For a more accurate analysis, the areas of the PIXE peaks together with the backscattered particle spectrum are fitted simultaneously with DataFurnace (see supporting information). The backscattered spectrum consists mainly of light elements (note the log scale) where the carbon, nitrogen and oxygen peaks from the cells can clearly be seen. Minor elements of the cell (Na, P, S) and the elements in the NPs (Ti, O) including a combined REE signal (Gd, Er, and Eu) can also be seen. The quantification of the Ti and REE signals from two regions scanned is shown in table 1. It is worth noting here that chemical fixation and air-drying can wash out diffusible elements such as phosphorus, so the absolute values of these elements measured in the sample may be less than in an unfixed state [17]. However, we have treated the data so that phosphorus is only used as a reference element. That is to say, we take the value of phosphorus and titanium for a ratio to compare cell-to-cell variability—not an absolute concentration.

**Figure 3.**
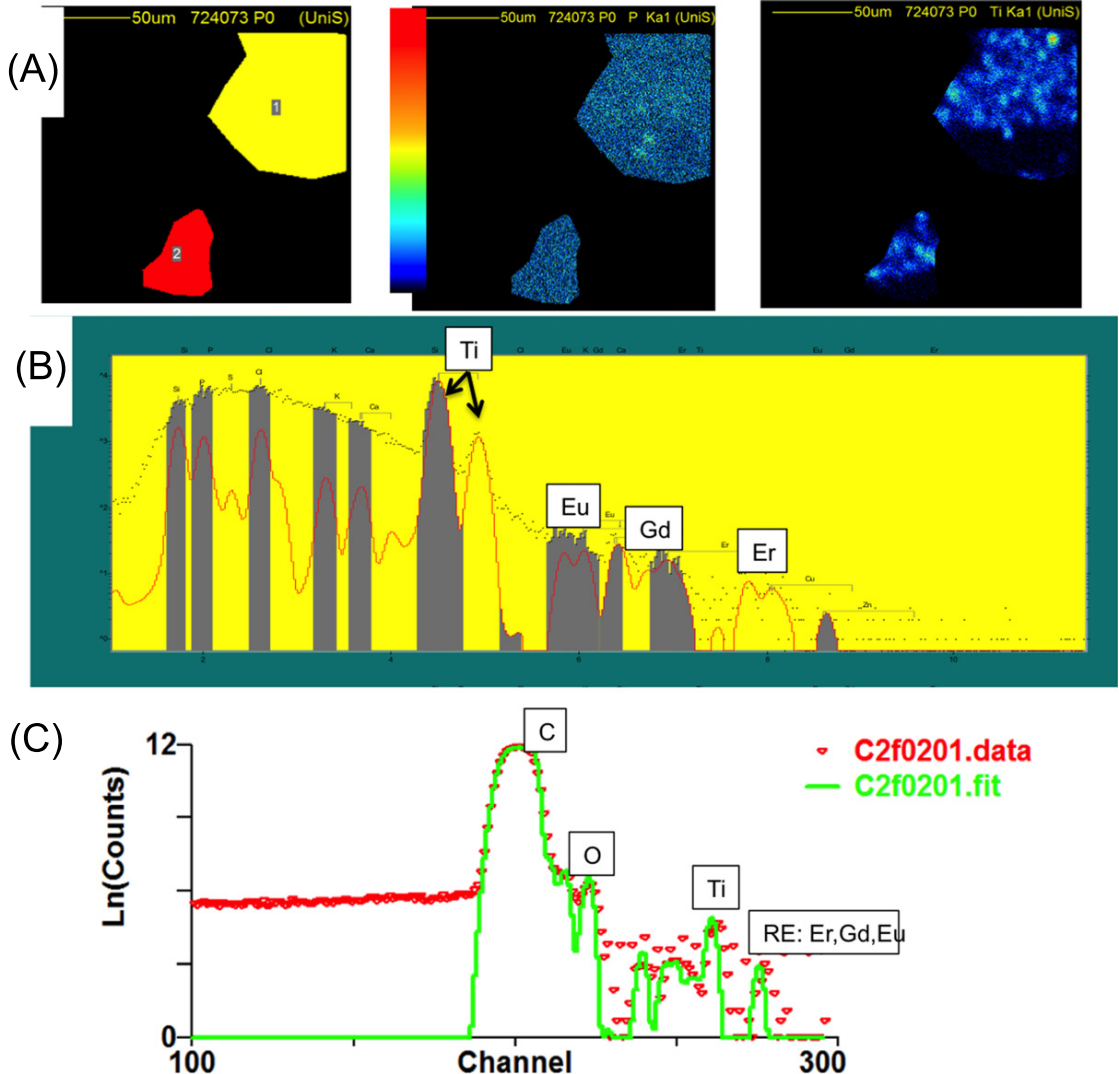
The figure shows the same sample as in figure 2, but here we have selected non-cell regions (A). (*left image*) A mask over areas where there are no cells. The phosphorus (*middle image*) and titanium signal (*right image*) show background levels of each element, and represent nanoparticles that have non-specifically bound to the fibronectin coated polypropylene substrate from the solution. The scale bar is linear and shows counts per pixel with a maximum of about 10^4^ counts (B). The proton induced x-ray emission (PIXE) signal of all the detectable elements of the masked regions. Note how the elements associated with cells (e.g. phosphorus, potassium) have almost undetectable peaks here, whereas titanium and rare earths signals are easily detectable (C). Elastic Backscattered particle spectrum (EBS) of the same region.

Figure 3 shows the same region as shown in figure 2, but we have selected areas where no cells are present. Here, we are able to analyse NPs that have non-specifically absorbed to the fibronectin coated polypropylene substrate, allowing a comparison between NPs that have been internalised inside the cells, to NPs that have not (it is important to note here that the polypropylene substrate is x-ray grade quality and gives no detectable signal other than the carbon from which it is composed). The data are processed in the same way as shown in figure 2. The PIXE spectrum shows clear Ti and REE signals, whereas the elements present in cells (such as phosphorus) have negligible peaks. The EBS spectrum shows clear Ti and REE signals, as well as carbon (from the polypropylene substrate), and some other peaks such as oxygen probably associated with proteins non-specifically absorbed to the fibronectin/substrate from the cell media, and from the oxide layer on the titania NPs themselves.

**Figure 4.**
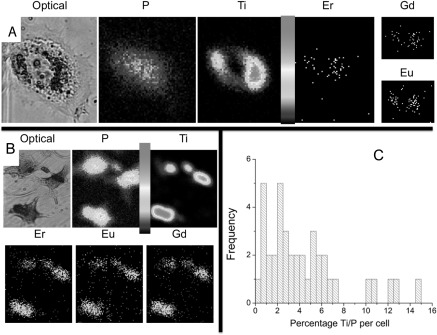
The location is shown of the NPs within the cells, and cell-to-cell variation of concentration of NPs in cells (A). An optical image shown alongside elemental maps of the same cell. Here, the NPs have localised around the nucleus (but not entered it), and the rare earth signals co-localise with the titanium signal (B). Example of the variation in the quantity of NPs in individual cells. From these images it is clear that each cell has a different concentration of Ti and REE within it compared to its neighbour. The scale bar in both A and B is linear and shows counts per pixel with a maximum of about 10^4^ counts (C). This variation was quantified in individual cells (*N* = 40) by using the Ti/P ratio from the PIXE signal and plotted as a histogram.

Table 1 shows the quantification of Ti and REEs from the EBS spectra comparing NPs inside and outside cells, in two different scanned samples (designated #77 and #73). There is a relatively large uncertainty of the REE quantification as there are lower counts and overlaps both between the REE L-line groups themselves and also with transition metal K-lines (such as Fe). However, when the NPs inside and outside cells are compared within the same region scan, the Ti/REE ratios when normalised are 1.00 and 1.05 and for the #77 and #73 samples respectively. This shows there is no significant difference between the REE content of NPs inside and outside the cells. As to the two samples having different ratios (84 compared to 65): it is possible that the counts obtained with the REE (around 80–200 counts per region) give sampling uncertainty—i.e. from region to region there is some difference due to counting statistics. This is why it is very important to compare NPs in cells to NPs on the substrate within the same region—and when this is done as is shown there is very little difference. Figure 4(a) shows that the Ti signal and the Er/Gd/Eu signal co-localise within individual cells. Here, a phase contrast image of an individual cancer cell is shown alongside the signal collected from the proton microprobe elemental imaging of the same cell. The outline of the cell can be seen showing more contrast in the cytoplasm and the nucleus with probably two nucleoli at its centre. Interestingly, a denser black area can be seen surrounding the nucleus. This dense area correlates extremely well with the strong titanium signal gathered from the PIXE spectra and associated maps. Moreover, this titanium signal correlates with the weaker Er/Gd/Eu signal. The peri-nuclear accumulation of NPs indicates that the NPs do not pass through the nuclear pores into the nucleus.

Figure 4(b) shows that the titanium and Er/Gd/Eu signal co-localise within each cell, but from the images it is clear that each cell contains different quantities of Ti and REE. To quantify this variation, each individual cell is delineated using OMDAQ software and an individual PIXE and backscattering spectrum is obtained for each cell. The PIXE spectrum is fitted and the ratio between the titanium and phosphorus signal is used to normalise the relative levels of titanium in each cell. Figure 4(c) shows a histogram of the Ti/P percentage in 40 individual cells. Some cells have more than 10 times the Ti/P than others, which is consistent with our previous findings with gold NPs [13] and confirming there is considerable heterogeneity in uptake within the population.

## Discussion

In this paper, we have used a proton microprobe to investigate the integrity of REE-doped titania NPs in individual human cells. We have shown that the titanium and REE signals co-localise, and that the Ti/REE ratio is no different inside and outside cells. This is important as it means that these NPs keep their integrity inside human cells *in vitro*, with an increased likelihood that they will also remain intact *in vivo*.

Using a proton microprobe is a powerful technique to probe elements in cells. However, because the REEs were at a very low concentration, acquisition time on each region of the sample was long (5 h) and counts on the REE remained relatively low (100–200 counts per cell). Coupled with limited accelerator access time and complicated analysis we were limited to the numbers of samples that could be analysed. Nevertheless, there are very few other techniques that could quantitatively detect dopant levels of REE in individual whole human cells.

There have been a few studies investigating the potential disintegration of NPs, either *in vivo* or *in vitro* [18] investigated CdSe quantum dots in acidic conditions to simulate the environment of the stomach or lysosomes (compartments in the cytoplasm of cells where NPs are sequested). They showed that acidic conditions could strip the NPs of PEG biocompatibility proteins and dissociate the NPs, releasing toxic Cd^2+^ ions. Similarly [19], showed that cobalt oxide NPs dissociated in the lysosomes, releasing solubilised cobalt ions. This increased the levels of reactive oxygen species in the cells ultimately resulting in cell death.

A further experiment [20] measured the ratio of Cd and Te in various organs of mice after injecting them with uncapped CdTe quantum dots. They found that from the 6 h timepoint onwards Cd was found in the liver and spleen, while Te was found in the liver. The conclusion was that the QDs did dissociate *in vivo* and that the free metals ions could be associated with toxicity.

There have been fewer studies on REE-doped NPs. However, REE toxicity is becoming increasingly important as their use in a range of industries increases. They are now commonly included in many products including magnets, electronics, mechanics and fuel additives [21]. This increase in demand has led to increased worker exposure in factories and mines. There are also concerns about the health risks of REE exposure since reports show lung fibrosis in polishers and rare earth mining workers [22], as well as systemic fibrosis in patients administered gadolinium based MRI agents [23].

The NPs used in this work are capped with silica. It is well known that capping with silica enables the NPs to become biocompatible, as the shell is hydrophilic and allows the covalent or ionic attachment of various proteins [24]. Capping NPs with a protective barrier such as silica is likely to maintain their integrity, prevent toxicity and prolong functionality [25]. It is possible that the combination of a silica layer and titania’s general structural robustness, allow for the NPs to maintain their integrity in the cells.

We have also shown a large variation in the numbers of NPs that enter individual cells within a population. This confirms our previous findings with gold NPs that there is more than a 10 fold difference in the concentration between individual cells [13]. Similar heterogeneity has been seen in analysis of single cells by a variety of different techniques and cell lines [9, 10, 26] including proton microprobes [27, 28]. This has important clinical applications for NPs use as therapeutics, especially when the localisation within the cell is considered [29]. NP concentration heterogeneity across cells in tissue could lead to an under-dosing of a subset of cells, which could potentially lead to resistant populations.

## Conclusions

Our results show that there is negligible difference between the composition of NPs inside and outside the cell—after 4 h of incubation. We have not performed a direct compositional comparison with the as manufactured NPs before they were incubated with the cell/medium. But due to the interstitial binding of the rare earths within the matrix of the titania, we believe it is unlikely that REE would leak out in a physiological buffer. It is much more likely that an acidic environment such as a lysosome inside a cell would break down the NP, but here we have shown that this almost certainly does not occur. Whilst in this instance it was important to determine that the REE did not leach from the titania, due to contra-indications of gadolinium associated nephrotoxicity, this work has wider implications for assessing other NPs which may be susceptible to leaching toxic ions. Furthermore, the assessment of the number of NPs per cell, and the associated heterogeneity, may prove useful for NP design or for the determination of NP dose regimes clinically.
